# Assessing child satisfaction and expectations for developing a child-friendly environment at the pediatric department in a general hospital in Qatar

**DOI:** 10.3389/fped.2024.1279033

**Published:** 2024-05-07

**Authors:** Amudha Pattabi, Ananth Nazarene, Sejo Varghese, Samiya Mohamud Hassan, Abdulqadir J. Nashwan, Surekha Kiran Patil, Kalpana Singh

**Affiliations:** ^1^ Nursing and Midwifery Education Department, Hamad Medical Corporation, Doha, Qatar; ^2^ Mental Health Services, Hamad Medical Corporation, Doha, Qatar; ^3^ Department of Nursing Education and Practice Development, Hazm Mebaireek General Hospital, Hamad Medical Corporation, Doha, Qatar; ^4^ Nursing and Midwifery Research Department, Hamad Medical Corporation, Doha, Qatar

**Keywords:** child satisfaction, child-friendly hospital environment, child expectation, children hospital, children

## Abstract

**Background:**

“Patient-centered” care positions the patient at the core and emphasizes fulfilling their unique needs, preferences, and values. This approach is particularly significant in the context of children. Although widely recognized as necessary, this approach is not universally implemented. The children find themselves in hospital wards where they are required to follow protocols and systems designed primarily for adults. In the appropriate atmosphere, children often express themselves more effectively through words, body language, and play, leading to a richer understanding of their needs. There is growing recognition of the importance of addressing children's concerns regarding hospital environments.

**Aim:**

This study investigates children's satisfaction with the physical aspect of the hospital environment. Insights from this exploration could provide valuable input for creating hospital environments centered around children's needs and preferences.

**Methods:**

This mixed-methods study involves children aged 6–14 years with parental consent from a premiere healthcare provider in the state of Qatar. The survey used nine items to gauge satisfaction with the existing hospital environment as a “child-friendly hospital” and another nine items to explore their expectations for such environments. The Mann–Whitney *U* and Kruskal–Wallis tests as well as thematic analyses were employed to assess the statistical significance of differences in satisfaction levels and children's expectations of the hospital's physical environment.

**Results:**

A total of 398 children participated in the study. Of them, 40.3% were aged 6–8 years; 60.3% had experienced two to five hospital visits; 55.8% of children participated during their outpatient service visit; and 31.7% were Asian. Children's satisfaction levels with various aspects of the hospital environment—including its physical appearance, signage, lounge, consultant rooms, corridors, bedrooms, TV content, toys, and staff uniforms—were in the range of 42.9%–59%. The children expressed a desire for a hospital environment that is spacious, colorful, attractive, and filled with cartoon characters and toys in the children's hospital from the front lounge to the inpatient units.

**Conclusion:**

The findings underline the importance of considering the perspectives of children in evidence-based healthcare design. The study reveals that children's satisfaction with the hospital environment is generally average or below average. Ultimately, a “child-friendly hospital environment” integrates children's rights into healthcare to significantly improve outcomes.

## 1 Introduction

General hospitals are built to meet the expectations of the population they are designed to serve (1). In the context of children, a vulnerable population with complex health needs (2), their preferences are seldom asked during significant decision-making processes (3). Moreover, children are reticent (4) and overwhelmed with fear and anxiety during their hospital visits because of the unfavorable environment (5).

Hospitals are designed per experts’ plans based on the provisions and perspectives of staff and policymakers (6). Incorporating patients’ views is vital while designing a hospital (7). The hospital visit rate for children is comparatively higher than that of other population groups (8); however, children's perspectives on how the hospital environment should be are often ignored (9) or the least heard (7), despite their greater sensitivity to the hospital environment compared to other populations (10).

Despite their significant differences from adults both mentally and physically (11), children are often provided with the standard hospital environment (12), yet children are uniquely sensitive to the hospital environment in a way that distinguishes them from adults (10). For example, they often experience stress during their hospital visits, and if any medical procedure is involved, their stress levels have been reported as high (13). According to Boucher et al. (14), play is a highly valued therapy for resolving these hospitalization-related problems. However, it is infrequently used during hospital visits or short stays due to its limited age range of application for children. In addition, the dynamic healthcare environment poses a significant challenge to the uniform implementation of play therapy (15).

“Patient-centered” care has introduced challenges to healthcare providers. This transformative approach places the patient at the forefront, focusing on meeting individual needs, preferences, and values (16). Planetree prioritizes crafting an outstanding environment for children, aiming to bolster their sense of security (17). Although unconventional, this approach is widely recognized for its effectiveness (18). When children are provided with a friendly physical environment, they communicate effectively through words, body language, and play; this in turn aids clinicians in understanding the children (14) and enables them to deliver care that is better received and accepted. In the hospital environment, children are timid, unwell, and quiet, and they can develop anxieties about the hospital atmosphere (12, 19). Incorporating children's input in shaping their hospital environment is vital (20); however, children are often overlooked (9), even if research has investigated children's hospital experiences for decades (7). A child-friendly environment must take into account various factors, such as sound (21), color (22), artwork (23), lighting (24), furniture (25), green spaces, conditions that allow for the presence of the children's families (26), and the atmosphere (27). Although much research has focused on patient satisfaction in hospital settings (28–30), few studies offer in-depth insight into hospital physical environments (31).

Though the “baby-friendly hospital” is a well-known concept around the world (32), a “child-friendly hospital environment” is an emerging concept and has not been fully explored (26). The current generation of children has numerous demands regarding their hospital stays (8). Several studies have explored parents’ expectations (33), concerns (34), perceptions (35), and needs (36) regarding their hospitalized child, but children have been relatively underrepresented in assessments of the same areas (8). When it comes to the physical environment of an institution, though the institution is designed for them, children's voices are often unheard.

Hamad Medical Corporation (HMC) is the principal public healthcare provider in the State of Qatar. Hamad General Hospital (HGH) provides highly specialized and complex care, including child healthcare. The aim of the present study was to learn directly from participants about HMC’s physical environment and their expectations for the features of the facility they use. This study strives to catch a glimpse inside the children's reality to determine what they need. Current research provides evidence of what characterizes the best healthcare service in Qatar (37, 38), and the authors firmly believe that even minor changes in the delivery setting can further enhance healthcare services. This is a foundational step in transitioning from traditional pediatric environments to child-friendly hospitals so that “best healthcare service” is not just a label but a reality.

## 2 Methods

### 2.1 Design, setting, and participants

This study uses a mixed-methods, convergent parallel design and includes children who visited/were admitted to the pediatric (pediatric inpatient medical and surgical unit, day-care, and outpatient unit) facility of HGH during the study period. Children were aged 6–14 years and required consent from their parents to participate.

### 2.2 Measures

The authors surveyed the pediatric facility (pediatric inpatient medical and surgical unit, day-care, and outpatient unit) in HGH during the study period. Both the qualitative and quantitative questionnaires were self-reporting questionnaires. In the qualitative questionnaire, the participants were expected to write their opinions and views on the listed questions.

The questionnaire contained three sections: section I consisted of seven questions regarding participants’ demographic details; section II consisted of nine questions to assess the level of satisfaction with existing hospital settings in terms of being a “child-friendly hospital” environment; and section III consisted of nine questions to determine participants’ expectations for a “child-friendly hospital.”

In section II, a self-reported questionnaire was adapted from another study (39) and duly prepared (in English and Arabic) by the researchers to assess the level of children's satisfaction with the hospital environment. The responses use a 5-point Likert scale score ranging from 0 to 5 (very poor to excellent).

In section III, a self-reported questionnaire was adapted from another study (39) and duly prepared (in Arabic and English) by the researchers for this study. Participants were asked to write down their opinions and views on the listed questions to collect qualitative data (40).

The section II and section III questionnaire responses were prepared with simple language and smileys representing the 5-point Likert scale to facilitate the children's understanding.

### 2.3 Ethical issues and data collection

The Institutional Review Board (IRB) at HMC reviewed and approved the study with the study number MRC-01-21-779.

### 2.4 Sample size

A purposive sampling technique was used to harvest the study samples. The sample size for the survey was calculated with a 95% confidence interval (CI) and a 5% margin of error. The authors enrolled 398 participants for the quantitative survey (sections 1 and II). In addition, a qualitative questionnaire was completed by 30 participants (section III).

### 2.5 Statistical analysis plan

The data were analyzed using Statistical Package STATA 17.0 software. Quantitative data were elegantly presented using the median [interquartile range (IQR)], while categorical data were conveyed with clarity, expressed as numbers and percentages. The Mann–Whitney *U* and/or Kruskal–Wallis tests determined the association between demographic variables. *p* < 0.05 was considered statistically significant.

During the initial steps of coding and categorization of the qualitative data, two researchers (AN and SV) evaluated the qualitative data independently. Any discrepancy in coding or categorization was resolved by discussion between the two researchers. These steps were employed to enhance the validity and reliability of the thematic analysis. The thematic analysis in this study was inductive and driven by participant data. The thematic analysis focused on extracting and understanding the critical aspects of the hospital environment that impact children's satisfaction based on their direct feedback and perspectives. This approach aligns with the principles of patient-centered care and provides valuable insights for designing child-friendly hospital environments.

The analysis identified five key themes representing children's preferences for hospital environments: (1) colorful and attractive—emphasizing vibrant colors for visual engagement; (2) pictures and cartoon characters—highlighting the need for familiar, entertaining visuals for comfort; (3) spacious and big—indicating a preference for ample space associated with freedom and comfort; (4) toys—underscoring the importance of play and entertainment to engage and distract children; and (5) clinicians’ uniforms—emphasizing the impression of clinicians is crucial for children in establishing a therapeutic alliance.

## 3 Results

### 3.1 Sociodemographic characteristics

A total of 398 children took part in the study. Of them, approximately 40.3% were aged 6–8 years, 33.9% were aged 9–11 years, and 25.8% were aged 12–14 years. Approximately half of the children (51%) were girls; 44.7% were in grade 1; 27.5% were in grades 4–6; and 27.8% were in grades 7–9. More than half of the children (60.3%) had visited the hospital 2–5 times, 29.9% were making their first visit, and 9.9% had made more than five visits. Of the children, 55.8% used outpatient services, 37.9% used inpatient medical services, and 6.2% used inpatient surgical services. Of the children, 41.3% were the firstborn in their family, 35.3% were the second born, and 23.5% were the third born or more among their siblings. Regarding nationality, 22.1% were from the Middle East and North Africa (MENA) region, excluding Qatar, 19.6% were Qataris, and 31.7% were Asian (Table 1).

**Table 1:** Sociodemographic characteristics of participants.

Variables	Level	Frequency (%)
		*N* = 398
Age (years)	6–8	159 (40.3%)
	9–11	134 (33.9%)
	12–14	102 (25.8%)
Gender	Male	194 (49.0%)
	Female	202 (51.0%)
Nationality	Asian	126 (31.7%)
	Qatari	78 (19.6%)
	MENA	88 (22.1%)
	Westerns/others	106 (26.6%)
Education	Grade 1–3	174 (44.7%)
	Grade 4–6	107 (27.5%)
	Grade 7–9	108 (27.8%)
Hospital visits	First visit	118 (29.9%)
	2nd–5th visit	238 (60.3%)
	More than fifth visit	39 (9.9%)
Sought service	OP service	215 (55.8%)
	Inpatient medical service	146 (37.9%)
	Inpatient surgical service	24 (6.2%)
Child order	First child	164 (41.3%)
	Second child	140 (35.3%)
	Third child	65 (16.4%)
	Four and above	28 (7.1%)

### 3.2 Child satisfaction with hospital's physical environment

The degree of satisfaction among children is displayed in Table 2. According to 33.2% of children, appearance was good/excellent, while 58.7% reported an average appearance. In addition, 32.3% of children agreed that signage was good/excellent, whereas 42.9% reported average satisfaction with signage. Furthermore, 26.5% agreed that the front lounge was excellent/good, and more than half thought it was average; regarding the consulting room, 33.5% felt it was excellent/good, while 50.6% thought it was average. A total of 31.7% thought the hallways were good/excellent, and 53.4% thought it was average. For hospital bedrooms, 27.5% said the bedrooms were excellent/good, whereas 59% described them as average. In addition, 60.0% of respondents thought the television content was average, while 22.3% said it was exceptional or good. In terms of toys, 57.4% of children thought the toys displayed were mediocre, while 21.7% thought they were great or good. Finally, 35.4% of children reported that the clinicians’ uniforms were excellent or good, and 52.5% reported them as average.

**Table 2:** Hospital environment satisfaction level.

Factor	Level	Value
*N*		398
Appearance	Very poor/poor	32 (8.1%)
	Average	233 (58.7%)
	Good/excellent	132 (33.2%)
Signage	Very poor/poor	98 (24.7%)
	Average	170 (42.9%)
	Good/excellent	128 (32.3%)
Front lounge	Very poor/poor	78 (19.7%)
	Average	213 (53.8%)
	Good/excellent	105 (26.5%)
Consultant room	Very poor/poor	62 (15.9%)
	Average	198 (50.6%)
	Good/excellent	131 (33.5%)
Hallway	Very poor/poor	59 (14.9%)
	Average	212 (53.4%)
	Good/excellent	126 (31.7%)
Bedroom	Very poor/poor	52 (13.5%)
	Average	227 (59.0%)
	Good/excellent	106 (27.5%)
Television content	Very poor/poor	69 (17.7%)
	Average	234 (60.0%)
	Good/excellent	87 (22.3%)
Toys displayed	Very poor/poor	81 (20.9%)
	Average	222 (57.4%)
	Good/excellent	84 (21.7%)
Uniform	Very poor/poor	48 (12.1%)
	Average	209 (52.5%)
	Good/excellent	141 (35.4%)

### 3.3 Qualitative findings for child expectations of hospital's physical environment

The children's responses on nine-item open-ended questionnaires were reviewed, and the preliminary data coding was done using deductive codes drawn from the research questions. The initial codes were grouped into five categories: (1) colorful and attractive; (2) pictures and cartoon characters; (3) spacious and big; (4) toys; and (5) staff uniforms (41).

#### 3.3.1 Colorful and attractive

##### P21: “colorful rooms that attract children and are decorated with cartoon characters”

Color can uplift, engage, calm, and heal, and children in particular might be more susceptible to the effects of color (42). In this study, children expected almost everything to be colorful, including the walls of the hospital, chairs in the lounge, designs in the hallway, examination beds in the consulting room, beds and bedsheets in the inpatient room, and colorful lights throughout the hospital. Creating a child-friendly and welcoming environment in hospitals is essential to helping ease their anxiety and empower them (43). When children see vibrant colors and fun designs, it can distract them from the medical setting, reduce stress, and create a sense of comfort. Children across cultures appreciate color, though color preference is multifactorial. In this study, children expressed that the colors they expected in hospitals were blue and pink. Though there are gendered associations with these two colors, their calming and relaxing properties should not be ignored (42). Children anticipated an appealing hospital lounge featuring a green garden, expansive play area, and engaging elements, such as balloons, cartoons, rhymes, and simplified health educational videos on television. They also desired a welcoming atmosphere with staff offering simple candies.

#### 3.3.2 Pictures and cartoons

##### P44: “child-friendly atmosphere with cartoon characters or animal pictures on the wall”

Children are very excited about pictures and cartoons, which are vital to their cognitive development (44). Studies have revealed the distractive abilities of photographs and cartoons, and this can therefore benefit children during their hospital visits (45). In this study, children shared their expectations that the hallway, waiting room, and even the consulting and inpatient units have pictures of cartoon characters. They added that hospital signage should have animal or cartoon characters and smileys, and they asked for children's drawings to be displayed on the hospital walls.

#### 3.3.3 Spacious and bigger

##### P90: “more spacious, more spacious…”

Children need space to walk and run as their imaginations are vast (46). For children, space is seen as an area to play, and the children expressed the same sentiment in this study. They did not want the hospital to look congested or crowded; they expected it to be spacious. Moreover, they expressed interest in a play area in the lounge or waiting room. Another concern was the size of the televisions; they wanted them to be big and play children's entertainment content in English or with subtitles. Even the signage is prominent, with animated letters on the cartoon characters and animals. Space is a significant concern for the children, especially in the waiting room (47). They also shared their thoughts on the chairs and noted that they would be happy if they were bigger and child-friendly, as opposed to traditional hospital chairs that make the environment look like a frightening emergency department.

#### 3.3.4 Toys

##### P77: “there should be a designated playroom where children can play and interact inside the room, and toys should be available for all and age-appropriate”

Toys significantly impact children's lives. It is therefore essential to consider this fact when choosing toys (48). Hospitals are unpleasant for children, and toys are the primary means of overcoming those negative perceptions and winning their confidence (49). The participants in this study reflected this notion. They expected toys at the front desk, in the hallway, hanging on the wall, in the signage, in the consulting room (rather than needles and other frightening medical devices), and in the hospital bed. In addition, they asked for age-appropriate, soft, educational, and interactive toys during their hospital stay. The consideration of children's perspectives on toys is essential, as the boost in a child's self-esteem and happiness, along with the facilitation of therapeutic relationships with health professionals, is achieved through toys, thereby benefiting the healing process (49).

#### 3.3.5 Clinicians’ uniforms

##### P7: “something more attractive and relaxing to relax children during their visits or stay, more stickers of different moods, cartoon characters, or interesting things they use in school”

When a nurse and a child first meet, the nurse's uniform and color scheme immediately cause a substantial amount of emotional upheaval. Uniforms are a non-verbal communication tool (50). The participants expressed that nurses’ uniforms bring needles to mind. They reported a preference for nurse uniforms that are pink or blue and have cartoons, flowers, or animals on them. While a white nurse uniform seems professional to adults, this attire is a source of fear, negative emotions, and anxiety for children and increases the perception of pain from treatments (51).

### 3.4 Factors associated with children's levels of satisfaction

The satisfaction score was determined by summing the answers to nine questions that gauged how satisfied respondents were with the current hospital settings as a “child-friendly hospital” environment. The total mean satisfaction score was 28.86 ± 6.81 (range 8–45).

Table 3 illustrates the relationship between demographic characteristics and children's levels of satisfaction. Children who received inpatient surgical treatment were more satisfied than those who received inpatient medical care (median 31.0; IQR 26.0–37.5) and outpatient (OP) services (median 27.0, IQR 25.0–36.0). In terms of the relationship between satisfaction level and age, sex, nationality, education, hospital visits, and child order, we did not discover any statistically significant differences (Table 3).

**Table 3:** Factors associated with children's levels of satisfaction and selected demographic variables.

Factor	*N*	Satisfaction, median (IQR)
Age (years)		
6–8	159	27.0 (25.0–33.0)
9–11	134	27.0 (24.0–34.0)
12–14	102	27.0 (25.0–33.0)
*p*-value		0.86
Gender		
Male	194	27.0 (24.0–32.0)
Female	202	27.0 (25.0–34.0)
*p*-value		0.2
Nationality		
Asian	126	27.0 (25.0–33.0)
Qatari	78	27.0 (24.0–35.0)
MENA (excluding Qatar)	88	27.0 (24.5–32.5)
Westerns/others	106	27.0 (25.0–33.0)
*p*-value		0.76
Education		
Grade 1–3	174	27.0 (25.0–33.0)
Grade 4–6	107	27.0 (25.0–36.0)
Grade 7–9	108	27.0 (24.0–29.5)
*p*-value		0.12
Visits to hospital		
1st visit	118	27.0 (25.0–33.0)
2nd visit	238	27.0 (24.0–33.0)
3rd visit	39	27.0 (27.0–35.0)
*p*-value		0.049
Service sought		
OP service	215	27.0 (25.0–36.0)
Inpatient medical service	146	27.0 (24.0–27.0)
Inpatient surgical service	24	31.0 (26.0–37.5)
*p*-value		<0.001^a^
Child order		
First child	164	27.0 (24.0–32.0)
Second child	140	27.0 (24.0–31.5)
Third child	65	27.0 (27.0–35.0)
Four and above	28	27.0 (25.0–33.0)
*p*-value		0.13

^a^ *p*-values were based on the Kruskal–Wallis test.

## 4 Discussion

Patient- and family-centered care is essential in pediatric healthcare systems worldwide (52). Patient satisfaction is significant in ensuring healthcare quality (53). The evolution of an efficient and long-lasting healthcare system depends on maintaining a patient-centered care model. Patient satisfaction and experience have traditionally been used to gauge healthcare quality (54). While studies on parental satisfaction in pediatric hospitals exist, they predominantly focus on the care perspective (55) rather than addressing the physical environment (56). Notably, children are often overlooked in expressing their experiences (57). Despite the traditional approach of surveying parental satisfaction, there is a growing trend to directly engage children in satisfaction surveys, recognizing their unique perspective (58).

Children can offer insightful comments about their experiences receiving medical care, and—more critically—children have a different perspective on receiving medical care than adults (59).

This study assessed satisfaction with the hospital's physical environment among children receiving medical services in the pediatric unit. A substantial amount of evidence supports that the physical environment of a healthcare facility has a more significant impact on treatment and recovery (60). Hence, all hospitals must strive to make their pediatric wings enjoyable and child-friendly (61).

Children's first impression of the hospital is crucial as it sets the tone for their entire stay. A welcoming and comforting environment from the outset is essential to ensuring a positive and satisfying patient experience right up until discharge (62). This study reveals that children's satisfaction with the hospital environment in the pediatric facility in this study is average regarding the hospital entrance, front lounge, and hallway appearance. The presence of parents during the data collection may explain this and could be perceived as a constraint to children sharing their negative views (63). The opposite trend was reported in a study from Taiwan, where a hospital environment featuring vivid, warm, and comfortable elements was added specifically for the children. It includes an open-air hanging garden, outdoor sculptures in the hospital's outer premises, a decorated waiting area, a donut-shaped lounge, turf, and warm sunlight in the hallway (64). The green, lush, nature-enriched hospital outer premises can counter the worry and fear of hospitals, as supported by decades of healthcare literature (65, 66). Regarding the physical setting, ventilation, lighting, and acoustics all proved significant for overall satisfaction (47). Children's expectations for a child-friendly hospital environment include a green garden with play facilities; a spacious, attractive lounge with surprise rewards; colorful chairs; and toys that facilitate meaningful engagement and distract from fear and anxiety.

Most children in this study reported average satisfaction with the hospital's physical structure and designs, and the same trend emerged regarding the content played on televisions placed in the waiting area, consulting room, and inpatient rooms. This indicates that participants were neither satisfied nor dissatisfied, and insistence on displaying cartoon movies plays a vital role in diverting their attention away from their distress and pain (67). Participants’ expectations regarding the televisions in the children's hospital were that they should be big and play cartoons, educational content, and simplified child-friendly health information in English or with English subtitles. Echoing the value of such entertainment, a study in Turkey revealed that animated cartoons displayed during painful procedures effectively reduced distress and pain (68), and an Italian study came to the same conclusion (69). Indeed, several studies have revealed that video distraction is an effective method of reducing anxiety in hospitalized children, comparable to oral midazolam or parental presence (70).

The toys and activities in the study hospital playroom are meant for the children during their hospital stay (7). However, the participants felt average satisfaction with the toys displayed and the activities they experienced during their hospital stays. Moreover, they expected colorful, soft, interactive, cartoon character-based toys to be placed in almost every area of the hospital, from the front lounge to inpatient beds. A study from Iran strengthened the evidence of the impact of toys and playrooms on hospitalized children (71).

An uncomfortable sleeping environment might significantly impact children's experience in hospital (72); unless addressed, this may negatively impact both the child and the parent (73). Study participants expressed average satisfaction with the bedroom provisions; this may have been due to their expectations that the bed and bedsheets feature cartoon characters and to find age-appropriate toys in the bedroom. A study reported the negative impact on children’s satisfaction as the hospital bedrooms failed to attract children (47).

Past evidence has supported children's desire for themed and colored-patterned uniforms (50) for healthcare workers. In the present study, more than half of the children stated that their satisfaction was average, while 10% reported poor satisfaction and 30% reported good satisfaction. This may be because they expected clinicians’ uniforms to be colorful and include cartoon characters, animals, or flowers.

A Swedish study (74) reported that children's fear compromises their satisfaction in the consulting room. It also reported the need for paintings, various kinds of animals, funny things to talk about, puzzles, and riddles. The lack of these features in the consulting room in this study could be the reason for half of the participating children's average level of satisfaction.

Children's age and sex are reported to be statistically significant in their satisfaction with a hospital's physical environment (64). However, the other demographic variables were not proven to have significance in our study regarding satisfaction with the hospital's physical environment.

## 5 Limitations

Children have had limited and narrow attention in hospital experience surveys. However, this study is one of the few conducted in the Middle Eastern region. A traditional, adult-style questionnaire was used to collect data from the child participants; though this could pose a limitation, the authors used emoji icons for children to express their satisfaction levels. The presence of parents during the survey may play a significant role in more neutral responses than others. Studies have proven that parents have ambivalent attitudes toward considering children's opinions, which may have imposed pressure on the participants to complete the questionnaire faster. Nevertheless, using mixed methods to strengthen the quantitative findings is the strength of the study.

The results of this study promote child-centered research approaches and the development of age-appropriate survey tools, and the findings also encourage more studies worldwide focused on enhancing the quality of pediatric healthcare services.

## 6 Conclusion

Ideally, the perspectives of children and parents utilizing healthcare facilities should be integrated into the development of policies, design strategies, and healthcare management for children; this approach will facilitate the creation of hospital environments that are supportive and tailored to the needs of children and adolescents, minimizing reliance on adults’ assumptions about children's requirements. Furthermore, careful planning of the physical environment, including elements like lighting, color schemes, sound attenuation, adequate ventilation, and artwork, can significantly contribute to children's wellness and healing processes.

## Data Availability

The raw data supporting the conclusions of this article will be made available by the authors, without undue reservation.
